# Unfolded protein response pathways in stroke patients: a comprehensive landscape assessed through machine learning algorithms and experimental verification

**DOI:** 10.1186/s12967-023-04567-9

**Published:** 2023-10-27

**Authors:** Haiyang Yu, Xiaoyu Ji, Yang Ouyang

**Affiliations:** 1 Henan University of Traditional Chinese Medicine, Zhengzhou, 450046 Henan China; 2 Xiangya Hospital, Central South University, 87 Xiangya Road, Changsha, 410008 Hunan China

## Abstract

**Background:**

The unfolding protein response is a critical biological process implicated in a variety of physiological functions and disease states across eukaryotes. Despite its significance, the role and underlying mechanisms of the response in the context of ischemic stroke remain elusive. Hence, this study endeavors to shed light on the mechanisms and role of the unfolding protein response in the context of ischemic stroke.

**Methods:**

In this study, mRNA expression patterns were extracted from the GSE58294 and GSE16561 datasets in the GEO database. The screening and validation of protein response-related biomarkers in stroke patients, as well as the analysis of the immune effects of the pathway, were carried out. To identify the key genes in the unfolded protein response, we constructed diagnostic models using both random forest and support vector machine-recursive feature elimination methods. The internal validation was performed using a bootstrapping approach based on a random sample of 1,000 iterations. Lastly, the target gene was validated by RT-PCR using clinical samples. We utilized two algorithms, CIBERSORT and MCPcounter, to investigate the relationship between the model genes and immune cells. Additionally, we performed uniform clustering of ischemic stroke samples based on expression of genes related to the UPR pathway and analyzed the relationship between different clusters and clinical traits. The weighted gene co-expression network analysis was conducted to identify the core genes in various clusters, followed by enrichment analysis and protein profiling for the hub genes from different clusters.

**Results:**

Our differential analysis revealed 44 genes related to the UPR pathway to be statistically significant. The integration of both machine learning algorithms resulted in the identification of 7 key genes, namely ATF6, EXOSC5, EEF2, LSM4, NOLC1, BANF1, and DNAJC3. These genes served as the foundation for a diagnostic model, with an area under the curve of 0.972. Following 1000 rounds of internal validation via randomized sampling, the model was confirmed to exhibit high levels of both specificity and sensitivity. Furthermore, the expression of these genes was found to be linked with the infiltration of immune cells such as neutrophils and CD8 T cells. The cluster analysis of ischemic stroke samples revealed three distinct groups, each with differential expression of most genes related to the UPR pathway, immune cell infiltration, and inflammatory factor secretion. The weighted gene co-expression network analysis showed that all three clusters were associated with the unfolded protein response, as evidenced by gene enrichment analysis and the protein landscape of each cluster. The results showed that the expression of the target gene in blood was consistent with the previous analysis.

**Conclusion:**

The study of the relationship between UPR and ischemic stroke can help to better understand the underlying mechanisms of the disease and provide new targets for therapeutic intervention. For example, targeting the UPR pathway by blocking excessive autophagy or inducing moderate UPR could potentially reduce tissue injury and promote cell survival after ischemic stroke. In addition, the results of this study suggest that the use of UPR gene expression levels as biomarkers could improve the accuracy of early diagnosis and prognosis of ischemic stroke, leading to more personalized treatment strategies. Overall, this study highlights the importance of the UPR pathway in the pathology of ischemic stroke and provides a foundation for future studies in this field.

**Supplementary Information:**

The online version contains supplementary material available at 10.1186/s12967-023-04567-9.

Ischemic stroke (IS) results from the narrowing or blockage of blood vessels supplying blood to the brain, leading to localized cerebral tissue ischemia, hypoxia, injury, and necrosis and resulting in symptoms of neurologic deficits [1]. The World Health Organization estimates that annually, approximately 15 million individuals experience a stroke, with one third of these individuals succumbing to the disease and another third becoming permanently disabled, thereby placing a significant strain on both families and society as a whole [2]. Clinical management of IS aims to restore cerebral tissue perfusion, primarily through pharmacologic thrombolysis or vascular intervention [3]. Despite its efficacy, thrombolytic therapy is contraindicated for certain patients due to factors like age, genetics, and environmental conditions, leading to a rising annual rate of disability and mortality among those with IS [4].Exploration of the signaling pathways underlying the pathological mechanisms of ischemic stroke holds great potential to enhance the treatment and management of this debilitating disease. One such pathway of interest is the Unfolded Protein Response (UPR), a cellular defense mechanism triggered in response to stress-induced accumulation of misfolded proteins in the mitochondria. The UPR involves the upregulation of associated proteins, with the goal of restoring mitochondrial protein homeostasis [5].UPR is crucial for maintaining mitochondrial protein homeostasis during cellular stress. Moderate activation of UPR can effectively restore protein balance, serving as a protective mechanism at the early stages of stress. Conversely, excessive UPR activation can induce excessive autophagy within the mitochondria, culminating in apoptosis [6].

The application of bioinformatics analysis techniques, particularly those based on high-throughput sequencing technology, has revolutionized our ability to delve into the molecular mechanisms of disease occurrence and development [7–9]. As a core component of artificial intelligence, machine learning has shown great potential in a range of medical fields, including biomedical research, personalized medicine, and computer-aided diagnosis. In the context of ischemic stroke, machine learning has been employed to enhance various aspects of diagnosis and prognosis [10]. A limited number of studies have harnessed the power of machine learning and bioinformatics to investigate potential target genes in IS and their associated immune effects [11, 12].In the present study, we leveraged the power of bioinformatics and machine learning to gain new insights into the underlying mechanisms of ischemic stroke. By analyzing the human IS transcriptome dataset from the Gene Expression Omnibus (GEO) database, we aimed to identify key genes related to the unfolded protein response (UPR) pathway, a cellular stress response that has been linked to the development of ischemic stroke. We employed a combination of two machine learning algorithms, random forest (RF) and support vector machines-recursive feature elimination (SVM-RFE), to identify and analyze the immune effects mediated by the UPR pathway. Our goal was to provide a foundation for the diagnosis and treatment of ischemic strokes, with the hope of ultimately reducing the burden of this debilitating disease.

## Data and methods

### Downloading and collating data

The present study leveraged the vast repository of information housed in the Gene Expression Omnibus (GEO) database of the National Center for Biotechnology Information (NCBI) to access and analyze two transcriptome datasets pertaining to IS. Specifically, the datasets GSE58294 and GSE16561, consisting of 69 IS samples and 23 healthy control samples, and 39 IS samples and 24 healthy control specimens, respectively, were obtained and subsequently merged utilizing the "sva" package. The clinical information of the samples in the dataset can be found in Additional file 1: Table S1 and Additional file 2: Table S2.

### Expression pattern and significance of genes related to the UPR pathway

We conducted a differential gene expression analysis of the merged mRNA expression data using the “limma” package, setting a threshold of adjusted p-value [13]. The resulting expression differences were visualized through heat maps and volcano plots generated using the “pheatmap” package. We also performed a correlation analysis of the differentially expressed genes and plotted corresponding correlation heat maps.The impact of the UPR pathway on IS was assessed through a one-sample enrichment analysis using the “GSVA” package. The ssGSEA scores of the UPR pathway genes were compared between the IS and control groups using a Wilcoxon signed rank test. These results provided a foundation for exploring the role of the UPR pathway in IS diagnosis and treatment.

### Joint random forest screening of key genes by machine learning and model building

Two machine learning algorithms, SVM-RFE and random forest, were employed to identify key genes associated with UPR [14, 15]. The “caret” and “randomForest” packages were utilized, respectively, for SVM-RFE and random forest analysis. The SVM-RFE algorithm, implemented with the caretFuncs recursive feature selection and K-fold cross-validation, was utilized to identify the feature genes for IS patients. The original dataset was split into 1000 different combinations using the random forest algorithm, with each combination generating a binary recursive classification tree. Ultimately, a random forest was constructed from these 1000 combinations, and the classification results were determined based on the voting outcomes of the trees. The accuracy of the classification was assessed using the out-of-bag estimation error rate. Through cross-validation, the random forest model with the minimum out-of-bag error rate was selected as the final model. Subsequently, the selected disease feature genes from both algorithms were intersected, and a multivariable logistic regression analysis was performed. The "forestploter" package was utilized to produce a forest plot displaying the contribution of these genes to the disease, as well as to generate logistic regression predictions for individual samples. To assess the diagnostic accuracy of the key genes, the area under the receiver operating characteristic curve (AUC) was calculated using the “pROC” package [16]. The model was resampled 1000 times with the “boot” package, and the average AUC values, sensitivity, specificity, and confidence intervals were obtained. The “regplot” package was used to generate nomograms depicting the key gene columns. Additionally, clinical calibration curves and decision curves were plotted using the “rmda” and “ggDCA” packages, respectively. The “ggstatsplot” package was utilized to plot the correlation of key clinically significant genes.

### In two analyses of immune infiltration based upon model genes

We sought to unravel the intricate relationship between the model genes and immune cells. To this end, we leveraged the power of the "IOBR" package [17], which enabled us to calculate this relationship using two cutting-edge algorithms: the CIBERSORT algorithm and the MCPcounter algorithm. Furthermore, we utilized the "ggscatterstats" package to analyze and visualize the correlation between the key model genes and immune cells, providing deeper insights into this crucial aspect of the study.

### Clustering typing and the clinical significance of samples

We Utilized the “ConsensusClusterPlus” Package to Systematically Cluster Genes Related to the UPR Pathway in IS Samples, Based on Gene Expression Profiles [18]. A Heat Map Integrating Gene Expression Data and Clinical Information, Such as Patient Age, Gender, and Time Since Stroke, was Generated. The Differences Among the Clusters Were Analyzed and Visualized through Bar Graphs. The “ggstatsplot” Package Was Employed to Assess Variations Among the Different Clusters with Respect to Gender and Time Since Stroke. In Addition, The "ggboxplot" Package Was Utilized to Depict the Relationship between Distinct Clusters and Immune Cells, Inflammatory Factor Expression, Score prediction by logistic regression, and Age of Onset.

### Central gene set enrichment analysis of different groups

We utilized the R package [19] “WGCNA” to analyze the gene expression matrix of the distinct clusters, with the results visualized as a correlation between different clusters and gene modules. Higher correlation coefficients were interpreted as greater relevance of the module to clinical data. "fgsea" was employed to conduct an enrichment analysis of the gene module clusters, and the results were visualized with the “pheatmap” package. To provide a comprehensive view of protein profiles translated by various groups of gene modules, we employed the Proteomaps database [20].

### Validation of expression of model genes in the blood of IS patients

Blood samples (5 mL) were collected from hospitalized IS patients and healthy individuals undergoing physical examination at Xiangya Hospital, anticoagulated with EDTA, and sent to the laboratory within 2 h. In accordance with standard procedures, RNA was extracted from blood samples and subjected to quality control and reverse transcription. The resulting cDNA was then amplified using specific primers for RT-PCR. The internal reference gene GAPDH was used to calculate the relative expression levels of the target gene, based on the Ct values obtained from the RT-PCR reaction. Approval for the current study was granted by the Ethics Committee of Xiangya Hospital. The primer sequences are listed in Table 1. The clinical sample information is presented in Additional file 3: Table S3.

**Table 1:** The primers used in this study

Name	Primer	Sequence	Size
GAPDH	Forward	5 ′- TCAAGAAGGTGGTGAAGCAGG-3′	115p
	Reverse	5 ′- TCAAAGGTGGAGGAGTGGGT-3′	
ATF6	Forward	5 ′- TTGGAACAGGATTCCAGGAG -3′	166 bp
	Reverse	5 ′- CCATCAGGGCTTTGTCATTT-3′	
EXOSC5	Forward	5 ′- TCTCTCCTGGCCTGTTGTCT -3′	226 bp
	Reverse	5 ′- CTGAGTAGAGCCCCTTGGTG-3′	
EEF2	Forward	5 ′- TCTACGGGGTTTTGAACAGG -3′	177 bp
	Reverse	5 ′- GCCAGTGGTCAAACACACAC-3′	
LSM4	Forward	5 ′- ATGGGGAGACGTACAATGGA -3′	188 bp
	Reverse	5 ′- CACCTCCTCCTTGACCATGT-3′	
NOLC1	Forward	5 ′- GCAAAGAAGGCTGCTGTACC -3′	246 bp
	Reverse	5 ′- CTGGTTCTTTGGTGGCTCAT-3′	
BANF1	Forward	5 ′- GACAACCTCCCAAAAGCACC -3′	195 bp
	Reverse	5 ′- GTGTCTTTCAGCCATTCCCG-3′	
DNAJC3	Forward	5 ′- GCAGATACACAGATGCTACCAG -3′	157 bp
	Reverse	5 ′- TGTAAAACTTCAGAACAAACCCTAA-3′	

## Results

### An analysis of the UPR pathway-related gene expression in is patients

**Fig. 1:**
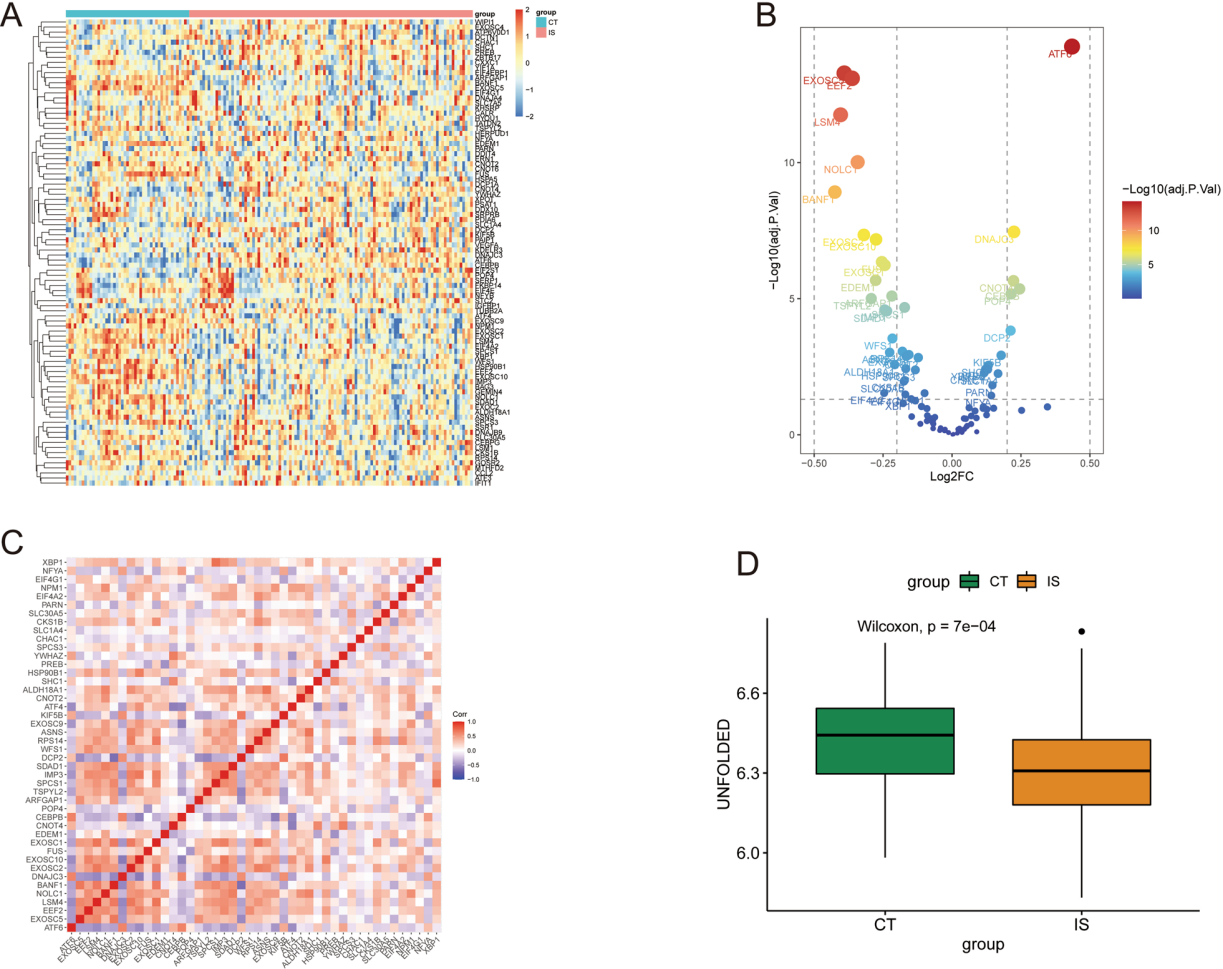
Expression of UPR Pathway-related Genes in IS Patients. **A**: Heatmap of UPR pathway-related genes. **B**: Volcano plot of UPR pathway-related genes. **C**: Heatmap of differential gene correlation in the UPR pathway. **D**: Difference in ssGSEA scores between the two groups

In this study, we merged two data sets and identified a total of 92 genes related to the UPR pathway. 41 of these genes, such as ATF6, DNAJC3, and CNOT441, were found to be upregulated in the IS cluster, while 51 genes, including EXOSC5, EEF2, and LSM4, were observed to be downregulated (Fig. 1A and B). Notably, 44 of the 92 UPR pathway-related genes were found to exhibit statistical significance after differential analysis. A correlation analysis showed that these genes were highly correlated with one another (Fig. 1C). These findings suggest that a single change in gene expression in the UPR pathway often leads to responses in multiple gene cascades. Additionally, the ssGSEA score in the IS group was significantly lower than that in the control group, indicating that the activation of the UPR pathway was significantly suppressed in IS patients (Fig. 1D).

### Joint random forest screening of key genes by machine learning and model building

**Fig. 2:**
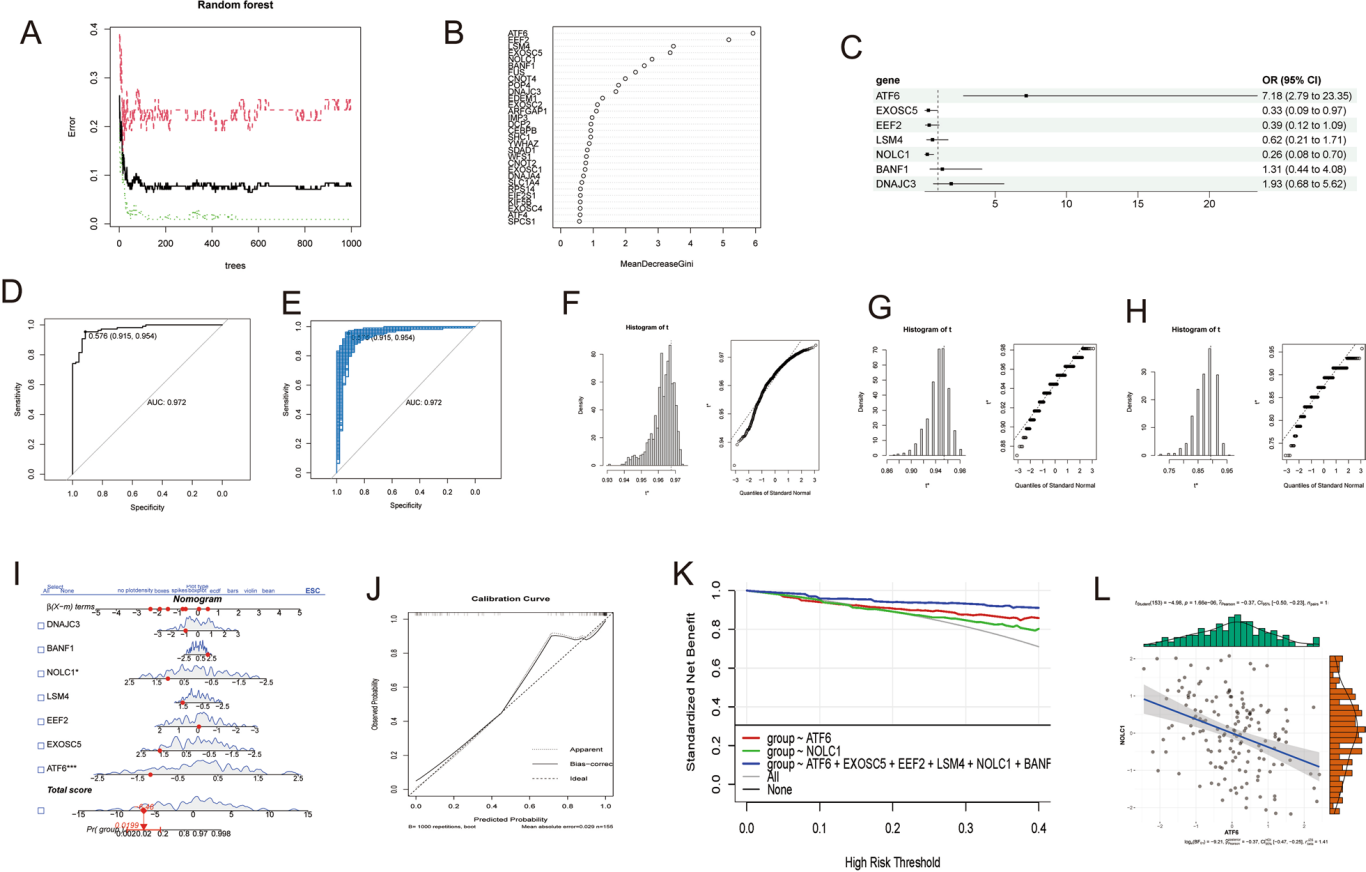
Machine Learning-assisted Random Forest Screening of Key Genes and Model Construction. **A**: Random Forest Fitting Plot. **B**: Top 30 Display Plot. **C**: Logistic Regression Plot. **D**: ROC Curve Plot. **E**: Bootstrap Random Sampling Plot. F) AUC Statistics Plot after Random Sampling. **G**: Sensitivity Statistics Plot after Random Sampling. **H**: Specificity Statistics Plot after Random Sampling. **I**: Norman Plot. **J**: Clinical Calibration Curve. **K**: DCA Clinical Decision Curve. **L**: Correlation Plot

We employed machine learning and random forest methods to screen the 92 genes related to the UPR pathway. Our analysis revealed 16 genes that were amenable to machine learning screening, and of these, 30 genes were subjected to further analysis (Fig. 2A, B). The goodness-of-fit plots generated by the random forest algorithm demonstrated its stability and high accuracy. Further analysis of the top ten genes from both methods led to the identification of seven key genes: ATF6, EXOSC5, EEF2, LSM4, NOLC1, BANF1, and DNAJC3. These seven genes were used in a multifactorial logistic regression analysis that revealed that ATF6, BANF1, and DNAJC3 may have elevated expression at the onset of IS, while EXOSC5, EEF2, LSM4, and NOLC1 may have decreased expression (Fig. 2C). The diagnostic model constructed from the seven key genes showed excellent performance, as indicated by the high area under the curve of the ROC curve, 0.972 (Fig. 2D). The model was tested on a random sample of 1000 patients and showed a mean value of approximately 0.963 (95% CI 0.9448–0.9718), sensitivity of approximately 0.945 (95% CI 0.9074–0.9722), and specificity of approximately 0.8757234 (95% CI 0.7872–0.9362) (Fig. 2F–H). When the seven key genes were plotted in a column line graph, we observed that ATF6 and NOLC1 had the greatest diagnostic significance for patients with IS (Fig. 2I). Further evaluation using clinical calibration and decision curve analysis demonstrated the stability and diagnostic performance of this column line graph model (Fig. 2J, K). Interestingly, our analysis also revealed a strong negative correlation between the expression of ATF6 and NOLC1, suggesting that these two genes may have opposing effects in patients with IS.

### In two analyses of immune infiltration based upon model genes

**Fig. 3:**
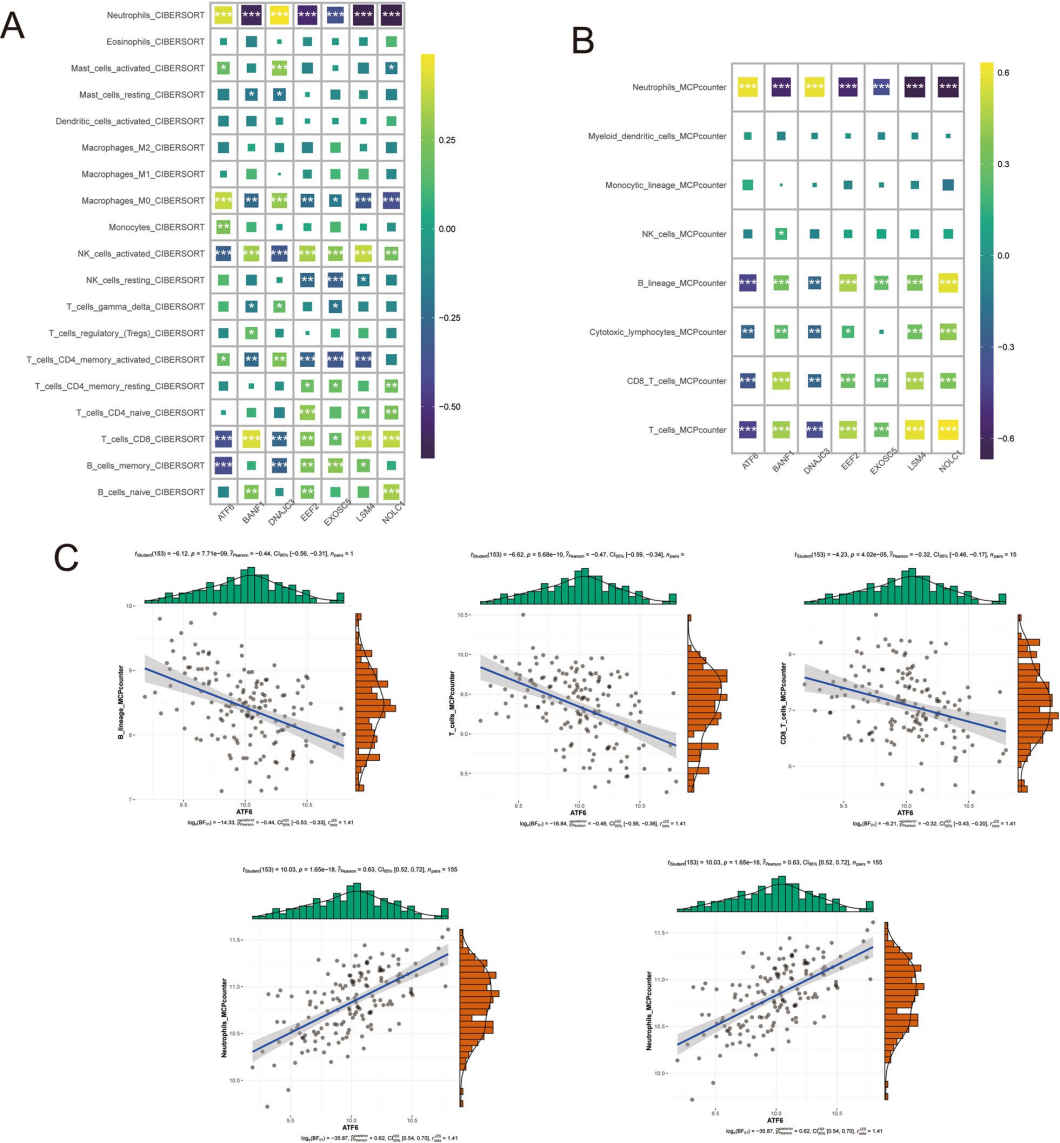
Immune cell infiltration analysis of model genes. **A**: Heatmap depicting immune cell infiltration using the CIBERSORT algorithm. **B**: Heatmap depicting immune cell infiltration using the MCPcounte algorithm. **C**: Correlation coefficient plot showing the association between ATF6 and immune cells

We used both the CIBERSORT and MCPcounter algorithms to examine the association of the seven key genes with immune cell populations in patients with IS. The results showed that all seven genes were strongly correlated with the infiltration of neutrophils and CD8 T cells (Fig. 3A, B). CIBERSORT also revealed that all seven genes were linked to macrophages (Macrophages_M0) and activated NK cells (NK_cells_activated). Further analysis using the MCPcounter algorithm revealed that these genes were also associated with the B cell lineage (B_lineage) and T cells (T_cells). These findings suggest that these genes play a critical role in driving the inflammatory response in patients with IS. Additionally, ATF6 was found to be highly correlated with multiple immune cell populations and was identified as the top weighted gene in the analysis (Fig. 3C).

### Clustering of sample typing and clinical significance

**Fig. 4:**
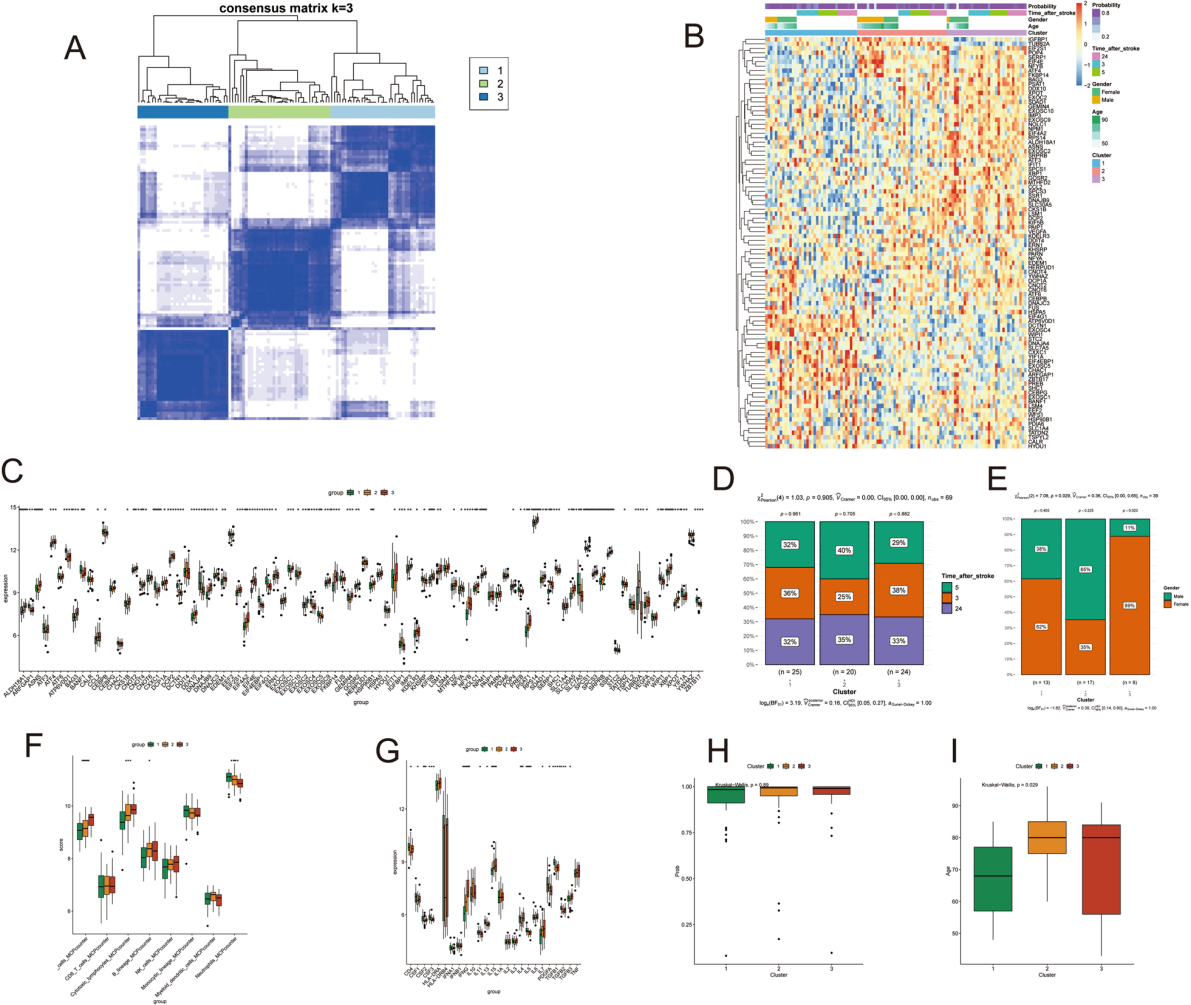
Cluster Typification of Samples and Clinical Significance.**A**: Heatmap of Consistency Matrix. **B**: Heatmap of Expression of UPR Pathway-related Genes in Different Clusters and Clinical Traits. **C**: Box Plot of Differential Genes in UPR Pathway. **D**: Distribution of Stroke Time in Different Clusters. **E**: Gender Distribution in Different Clusters. **F**: Immune Cell Infiltration in Different Clusters. **G**: Inflammatory Factor Secretion in Different Clusters. **H**: Differences in Logistic Regression Predictive Values in Different Clusters. **I**: Age Distribution in Different Clusters

Based on the clustering results, the IS samples were categorized into three distinct clusters (Fig. 4A). Notably, the expression profiles of UPR pathway-associated genes and clinical features differed significantly among the three clusters (Fig. 4B). Moreover, significant variations in gene expression levels, immune cell infiltration, and inflammation marker secretion were observed among the three clusters (Fig. 4C, F, G). Gender and age distribution of the patients were statistically significant among the three clusters (Fig. 4E, I). Importantly, the distribution of time after stroke and logistic regression prediction scores was largely consistent across the three clusters (Fig. 4D, H). By comparing the expression of UPR pathway-related genes in the three groups through the heatmap analysis, we observed that SLC7A5 exhibited the highest expression in cluster 1, TUBB2A in cluster 2, and PSAT1 in cluster 3. Therefore, these three genes can be considered as characteristic genes for each cluster. However, correlation analysis between these genes and clinical features such as age and gender revealed weak or no significant correlations. This suggests that the intervention of these clusters on clinical features may not be regulated solely by a single characteristic gene. The considerable variation in clinical features likely requires the comprehensive regulation of multiple genes to accomplish. These findings suggest that our proposed classification scheme not only has broad applicability to most post-stroke patients but also captures inherent molecular mechanisms underlying variations across genders and ages.

### Central gene set enrichment analysis of different clusters

**Fig. 5:**
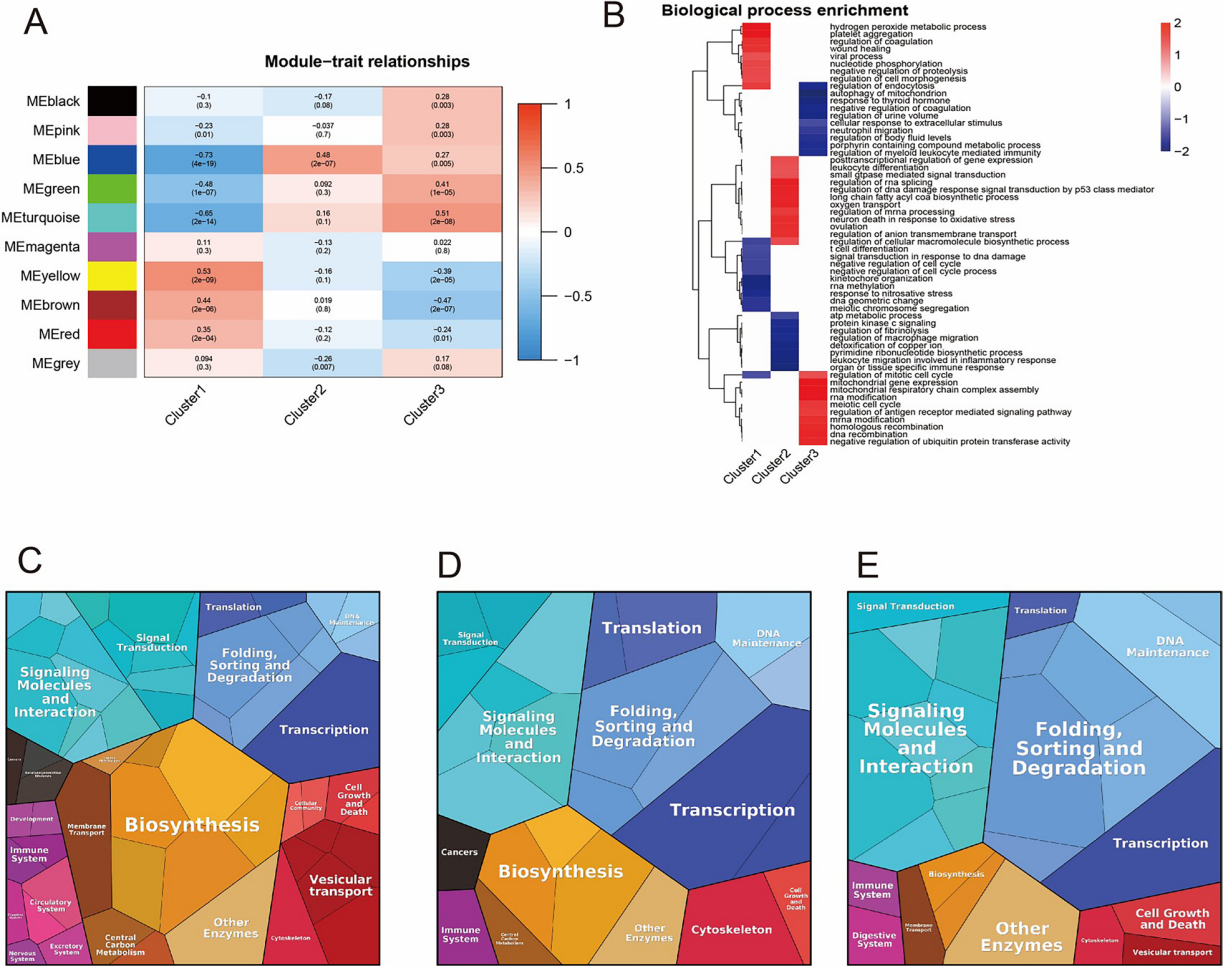
Cluster-Specific Core Gene Enrichment Analysis. **A**: WGCNA analysis of different clusters. **B**: FGSEA enrichment of different clusters. **C**: Protein landscape of Cluster 1. **D**: Protein landscape of Cluster 2. **E**: Protein landscape of Cluster 3

Building upon the results of WGCNA, we observed a correlation between different clusters and the “blue gene” module (Fig. 5A). Further analysis using fgsea revealed that the genes within Cluster 1 were primarily involved in processes such as platelet aggregation through hydrogen peroxide metabolism, regulation of wound healing, nucleotide phosphorylation, and the downregulation of cellular morphogenesis by protein hydrolysis and endocytosis (Fig. 5B).Cluster 2 genes, on the other hand, were predominantly linked to post-transcriptional regulation of gene expression in leukocyte differentiation, regulation of RNA splicing by small GTPase signaling, regulation of p53-like signaling in response to DNA injury, the biosynthesis of the second long chain fatty acyl CoA, and regulation of mRNA oxygen transport processing (Fig. 5B).Finally, Cluster 3 genes were found to be concentrated in processes such as regulation of the mitotic cell cycle, regulation of mitochondrial gene expression, modification of mitochondrial respiratory chain complex assembly proteins, and regulation of mRNA modification by antigen receptor-mediated signaling pathways (Fig. 5B).

Our results also showed a progressive increase in the proportion of patients involved in the folding, sorting, and degradation of proteins across the three clusters (Fig. 5C–E), which was found to be linked to the degree of activation in the UPR pathway.

### Validation of expression of model genes in the blood of IS patients

**Fig. 6:**
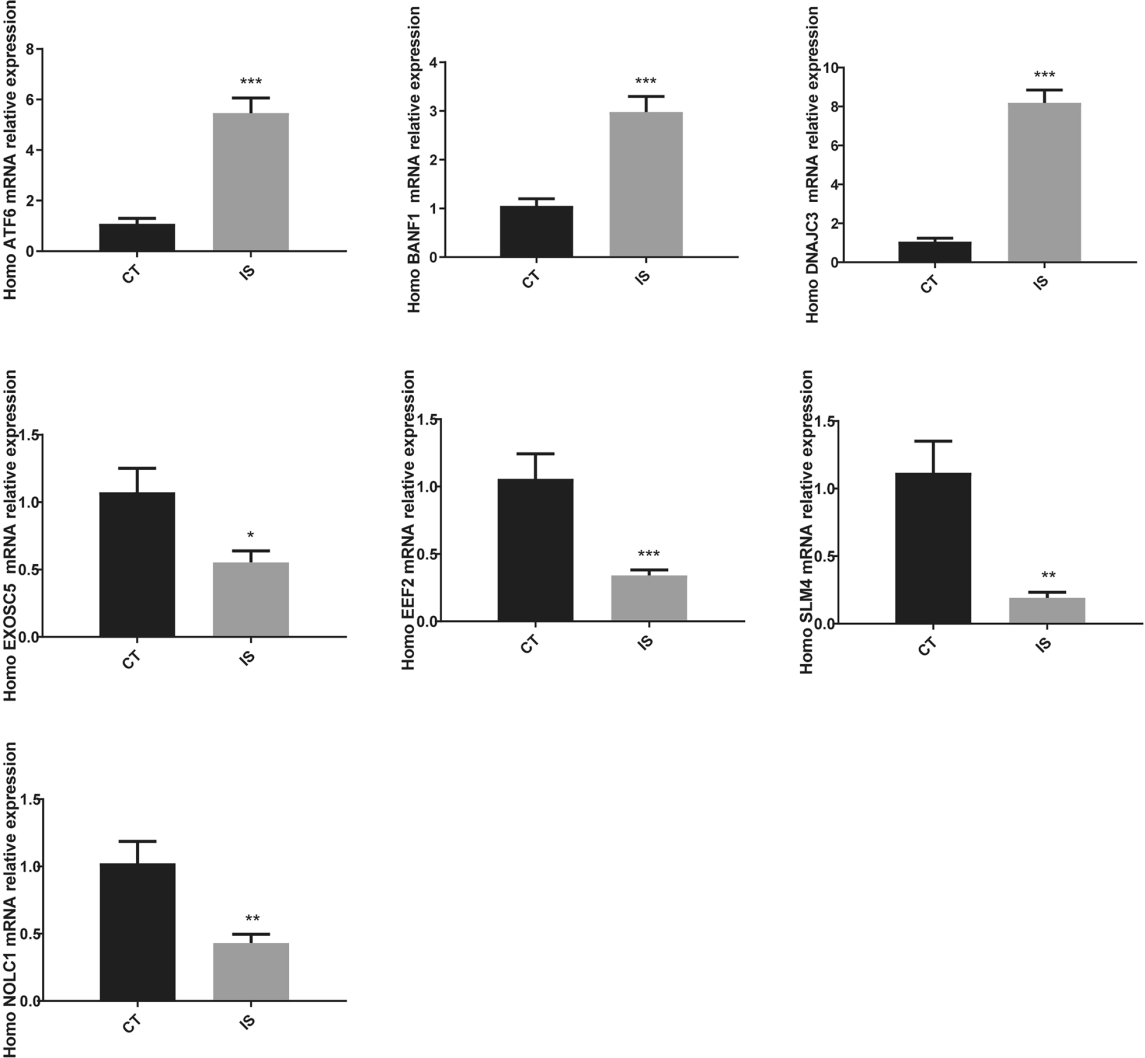
Validation of the target gene expression (n = 3). Compared with the control group, *P < 0.05, **P < 0.01, ***P < 0.001

The expression levels of model genes in the blood of IS patients and healthy individuals were detected and compared using RT-PCR (Fig. 6). The results showed that the expression levels of ATF6, BANF1, and DNAJC3 genes were upregulated in the blood of IS patients compared to healthy individuals (P < 0.05). Additionally, the expression of EXOSC5, EEF2, LSM4, and NOLC1 genes in the blood of IS patients was suppressed (P < 0.05).

## Discussion

Mitochondria, the central hub of cellular metabolism and energy production, play a crucial role in various physiological functions, such as apoptosis and immunity [21]. Maintenance of mitochondrial function is essential for cell survival and the proper functioning of the body. In response to stressors, such as glucose deprivation or glycoprotein glycosylation deficiency, cells can repair damaged mitochondria through a series of adaptive responses, including upregulation of protein synthesis and secretion, as well as failure of protein folding, transport, or degradation. UPR is a critical component of these repair mechanisms [22].UPR pathway serves as a critical mechanism to restore the balance of cellular homeostasis by upregulating the expression of mitochondrial molecular chaperones, heat shock proteins (HSP60, HSP70), and proteases [23, 24]. Upon activation, the UPR optimizes the flow of mitochondrial proteins into and out of the mitochondria through up-regulation of gene transcription and promotion of protein degradation via the ubiquitin proteasome. In instances where the UPR is unable to fully repair mitochondrial damage, it triggers mitochondrial fission to isolate the damaged region from the healthy mitochondrial network and removes structurally damaged mitochondria through phagocytosis. These mechanisms ensure that the integrity of the mitochondrial network is maintained, which is essential for the proper functioning of cells and the body as a whole [25].UPR and mitochondrial autophagy are two distinct mechanisms for repairing damaged mitochondria. While UPR dynamically regulates the degradation of mitochondrial proteins, mitochondrial autophagy results in the degradation of most mitochondria and a reduction in ATP production, ultimately leading to cellular death. In this sense, the UPR represents a more nuanced approach to maintaining mitochondrial homeostasis, fine-tuning mitochondrial behavior in response to stress.

Our research findings highlight the significance of genes associated with the UPR pathway, including ATF6, EXOSC5, EEF2, LSM4, NOLC1, BANF1, and DNAJC3, in the occurrence of stroke. Our study highlights the crucial role of genes related to the UPR pathway in the occurrence of stroke, specifically in the regulation of functional brain damage. Of these genes, ATF6, a membrane protein localized in the endoplasmic reticulum, has been shown to play a significant role in this process [26]. The activation of ATF6 in the brain after a stroke has been found to reduce functional brain damage, potentially through the promotion of UPR [27, 28]. This hypothesis is supported by studies in mouse models of cerebral ischemia, which reconfirm that ATF6 deficiency leads to more severe functional impairment and a worse prognosis, likely due to the inhibition of the protective effects of ATF6 against organ injury during ischemia [27]. It is evident that ATF6 activation represents a promising target in the search for therapies to mitigate the functional consequences of stroke. Exosc5, a component of the RNA exosome complex, is involved in numerous cellular processes related to RNA processing and degradation [29]. Importantly, mutations in the EXOSC5 gene have been linked to cardiac conduction defects, arrhythmias, and an increased risk of sudden cardiac death [30]. EEF2 is a vital player in the process of protein synthesis as a translation factor. It facilitates the transfer of tRNA from the A site to the P site of the ribosome via GTP hydrolysis, enabling the progression of tRNA along the ribosome's mitochondria and the extension of the peptide chain [31]. However, phosphorylation of EEF2 at threonine 56 by EEF2K has been shown to disrupt its ability to bind to the ribosome and participate in protein synthesis, leading to altered synaptic remodeling and impaired learning and memory functions [32]. These findings emphasize the crucial role of EEF2 in the regulation of protein synthesis and highlight the importance of further investigation into its cellular mechanisms.Sm-like4 (LSM4), a member of the RNA binding protein family, is a small nuclear ribonucleoprotein that has been linked to the rate of degradation of histone mRNA [33]. Strikingly, LSM4 methylation has been implicated in the formation of large arterial plaques, which is a known risk factor for stroke [34]. On the other hand, Nucleolar and coiled coil phosphoprotein1 (NOLC1) is a phosphoprotein that comprises a single core repeat structural domain and both N-terminal and C-terminal structural domains [35–37]. This protein is believed to play a role in various molecular processes, including DNA replication, amino acid metabolism, and expression of proteins involved in RNA processing [38]. Barrier-to-autointegration-factor (Banf1) is a small non-specific DNA-binding protein that plays a crucial role in maintaining nuclear membrane integrity and chromatin structure [39]. Banf1 has been observed to migrate from the nuclear membrane to sites of DNA damage, where it likely participates in the repair process [40]. In contrast, DnaJ Heat Shock Protein Family Member C3 (DNAJC3) is primarily found in the endoplasmic reticulum and acts to prevent misfolding of newly synthesized proteins by transiently binding a broad range of these proteins [41]. Interestingly, DNAJC3 has been shown to ameliorate lesions such as endoplasmic reticulum stress and neurodegeneration in mice, resulting in improved quality of life for patients [42]. These findings suggest that the body spontaneously activates genes related to the UPR pathway following stroke onset to counteract the subsequent harmful effects, underscoring the importance of further research into the molecular mechanisms of these genes. Finally, RT-PCR results demonstrated that the expression of these genes in the peripheral blood of IS patients was consistent with our study, providing further evidence of the clinical value of our research.

The expression of several genes was found to be linked to the infiltration of immune cells in the wake of ischemic injury. In particular, the degree of neutrophil infiltration was shown to have a positive correlation with the expression of genes such as ATF6 and DNAJC3, and an inverse correlation with the expression of genes like EXOSC5, EEF2, and LSM4. The number of neutrophils, in turn, is linked to various consequences of ischemia, such as infarct size, blood–brain barrier disruption, and neurological function [43, 44]. The release of chemotactic factors from the damaged tissue prompts the release of neutrophils from the bone marrow and their recruitment to the site of injury, accompanied by an increase in the expression of neutrophil adhesion molecules [45]. However, the large accumulation of neutrophils in blood vessels can lead to blockages and reductions in blood supply to the brain. Moreover, neutrophils may also bind to platelets through P-selectin glycoprotein ligand-1 or MAC-1, contributing to platelet aggregation and the formation of emboli [46].

Neutrophil recruitment to the site of injury can be motivated by the release of chemotactic factors and can result in the accumulation of large numbers of neutrophils in the blood vessels, leading to blockage and affecting blood supply to the brain [47]. The production of neutrophil extracellular traps (NETs) by neutrophils can promote coagulation and thrombosis, and high levels of the NET-specific marker circulating citrullinated histone H3 (citH3) have been associated with the development of atrial fibrillation and all-cause mortality in acute stroke patients.In addition to neutrophils, CD8 + T cells and NK cells are recruited within 24 h of a stroke, mediating the ensuing inflammatory response [48, 49]. B cells can also accumulate in the area of infarction and produce antibodies, which can contribute to cognitive impairment and affect the quality of survival for stroke patients [50].Taken together, these findings suggest that changes in the expression of UPR pathway-related genes may play a crucial role in the immune response to a stroke, affecting immune cell infiltration and, therefore, the outcome for patients.

In our latest study, we investigated the distribution of clinical traits such as gene expression related to the Unfolded Protein Response (UPR) pathway, as well as gender and age, in patients with ischemic stroke (IS). Our results showed some differences in these traits among IS patients in different clusters. Furthermore, gene modules from these patients revealed that the UPR signaling pathway was consistently enriched in all patient groups. Additionally, we observed variations in the protein folding, sorting, and degradation process among IS patients in different clusters.

To uncover the underlying molecular mechanisms of IS, we analyzed gene microarray data from both IS patients and healthy controls. Our analysis revealed key genes related to the UPR pathway, which were identified by two machine learning algorithms. Based on these findings, we were able to construct a diagnostic model with high sensitivity and specificity. Finally, we investigated the relationship between genes related to the UPR pathway and clinical traits, as well as their impact on immune responses in IS patients.

## Conclusion

In the present study, we explored the relationship between the expression of genes related to the Unfolded Protein Response (UPR) pathway and the clinical traits, immune cell infiltration, and inflammatory factor secretion in patients with ischemic stroke (IS). Our analysis of gene microarray data from blood samples revealed a close association between these factors. Moreover, diagnostic models built based on genes related to the UPR pathway, such as ATF6, EXOSC5, EEF2, LSM4, NOLC1, BANF1, and DNAJC3, demonstrated high applicability value in identifying IS patients. Moreover, these genes were validated for expression using RT-PCR, providing further evidence of their reliability. Particularly noteworthy was the observation that ATF6 was found to be highly correlated with multiple immune cell infiltrations. These findings provide valuable insights into the underlying mechanisms of IS and could inform the development of more effective diagnostic and therapeutic strategies for this debilitating condition.

Of course, our study has certain limitations. Firstly, the inclusion of a relatively small number of healthy and disease groups based on the GEO database may not fully cover all the disease features shared by IS patients. Secondly, despite utilizing two machine learning algorithms and employing bootstrap resampling to construct and validate the diagnostic model to minimize overfitting and selection bias, there may still be potential false negatives or false positives biases. In the future, our team will conduct large-scale, multicenter clinical studies to further investigate the changes in the UPR pathway within IS patients and its impact on clinical features.

### Supplementary Information

**Additional file 1: Table S1**. Summary descriptives table of GSE58294.

**Additional file 2: Table S2**. Summary descriptives table of GSE16561.

**Additional file 3: Table S3**. The clinical sample information.

## Data Availability

The data used to support the fndings of this study are included within the article.
