# Supplementary material for: Unfolded protein response pathways in stroke patients: a comprehensive landscape assessed through machine learning algorithms and experimental verification
Source: J Transl Med. 2023 Oct 27;21:759. doi: 10.1186/s12967-023-04567-9 (PMC10605787; doi:10.1186/s12967-023-04567-9)
Supplement: Supplementary file 1 — Additional file 1: Table S1. Summary descriptives table of GSE58294. [file 12967_2023_4567_MOESM1_ESM.doc]

Summary descriptives table of GSE58294

|  | **Control** | **Stroke** | **p.overall** |
| --- | --- | --- | --- |
|  | ***N=23*** | ***N=69*** |  |
| Time_after_stroke: |  |  | . |
| 3 hour | 0 (.%) | 23 (33.3%) |  |
| 5 hour | 0 (.%) | 23 (33.3%) |  |
| 24 hour | 0 (.%) | 23 (33.3%) |  |
| City: Sacramento | 23 (100%) | 69 (100%) | . |
