# Supplementary material for: Unfolded protein response pathways in stroke patients: a comprehensive landscape assessed through machine learning algorithms and experimental verification
Source: J Transl Med. 2023 Oct 27;21:759. doi: 10.1186/s12967-023-04567-9 (PMC10605787; doi:10.1186/s12967-023-04567-9)
Supplement: Supplementary file 2 — Additional file 2: Table S2. Summary descriptives table of GSE16561. [file 12967_2023_4567_MOESM2_ESM.doc]

Summary descriptives table of GSE16561

|  | **Control** | **Stroke** | **p.overall** |
| --- | --- | --- | --- |
|  | ***N=24*** | ***N=39*** |  |
| Age | 59.9 (9.73) | 73.1 (14.0) | <0.001 |
| Gender: |  |  | 1.000 |
| Female | 14 (58.3%) | 22 (56.4%) |  |
| Male | 10 (41.7%) | 17 (43.6%) |  |
| Race: Caucasian | 24 (100%) | 39 (100%) | . |
