# Supplementary material for: Unfolded protein response pathways in stroke patients: a comprehensive landscape assessed through machine learning algorithms and experimental verification
Source: J Transl Med. 2023 Oct 27;21:759. doi: 10.1186/s12967-023-04567-9 (PMC10605787; doi:10.1186/s12967-023-04567-9)
Supplement: Supplementary file 3 — Additional file 3: Table S3. The clinical sample information. [file 12967_2023_4567_MOESM3_ESM.doc]

Supplementary Table 3: The clinical sample information.

|  | **Control** | **Stroke** | **p.overall** |
| --- | --- | --- | --- |
|  | ***N=3*** | ***N=3*** |  |
| Age | 61.0 (1.00) | 61.3 (1.15) | 0.725 |
| Gender: |  |  | 1.000 |
| Female | 1 (33.3%) | 2 (66.7%) |  |
| Male | 2 (66.7%) | 1 (33.3%) |  |
| Race: The Han nationality | 3 (100%) | 3 (100%) | . |
